# The effectiveness of ω-3 polyunsaturated fatty acid interventions during pregnancy on obesity measures in the offspring: an up-to-date systematic review and meta-analysis

**DOI:** 10.1007/s00394-018-1824-9

**Published:** 2018-09-24

**Authors:** Mariam Vahdaninia, H. Mackenzie, T. Dean, S. Helps

**Affiliations:** 1 School of Health Sciences and Social Work (SHSSW), University of Portsmouth, James Watson West, 2 King Richard 1st Road, Portsmouth, PO1 2FR UK; 2 Faculty of Health and Social Sciences, Bournemouth University, Poole, UK; 3 Research and Enterprise, University of Brighton, Brighton, UK

**Keywords:** Systematic review, Meta-analysis, Fatty acids, Fish oil, N-3 LCPUFA, Obesity, Childhood obesity, Adiposity, Body composition, Growth, Infant’s growth

## Abstract

**Background:**

The potential role of ω-3 long chain polyunsaturated fatty acid (LCPUFA) supplementation during pregnancy on subsequent risk of obesity outcomes in the offspring is not clear and there is a need to synthesise this evidence.

**Objective:**

A systematic review and meta-analysis of randomised controlled trials (RCTs), including the most recent studies, was conducted to assess the effectiveness of ω-3 LCPUFA interventions during pregnancy on obesity measures, e.g. BMI, body weight, fat mass in offspring.

**Methods:**

Included RCTs had a minimum of 1-month follow-up post-partum. The search included CENTRAL, MEDLINE, SCOPUS, WHO’s International Clinical Trials Reg., E-theses and Web of Science databases. Study quality was evaluated using the Cochrane Collaboration’s risk of bias tool.

**Results:**

Eleven RCTs, from ten unique trials, (3644 children) examined the effectiveness of ω-3 LCPUFA maternal supplementation during pregnancy on the development of obesity outcomes in offspring. There were heterogeneities between the trials in terms of their sample, type and duration of intervention and follow-up. Pooled estimates did not show an association between prenatal intake of fatty acids and obesity measures in offspring.

**Conclusion:**

These results indicate that maternal supplementation with ω-3 LCPUFA during pregnancy does not have a beneficial effect on obesity risk. Due to the high heterogeneity between studies along with small sample sizes and high rates of attrition, the effects of ω-3 LCPUFA supplementation during pregnancy for prevention of childhood obesity in the long-term remains unclear. Large high-quality RCTs are needed that are designed specifically to examine the effect of prenatal intake of fatty acids for prevention of childhood obesity. There is also a need to determine specific sub-groups in the population that might get a greater benefit and whether different ω-3 LCPUFA, i.e. eicosapentaenoic (EPA) vs. docosahexanoic (DHA) acids might potentially have different effects.

## Introduction

The high prevalence of childhood obesity is a serious public health issue and has been reported as a potential risk factor for a range of morbidities and medical conditions that occur later in life [1–4].

The very early days of fetal development, representing a period of substantial developmental plasticity, are thought to impact on health in the life course [5]. The developmental origins of health and disease theory proposes that events/exposures during this period are a key determinant of the susceptibility to many chronic diseases, including metabolic disorders and obesity [6]. It is well documented that the foetus responds to external stimuli in utero and environmental factors such as maternal diet could have effects later in life [7–10]. In this context, interventions that target the nutritional adequacy of women’s diet during pregnancy could provide a unique opportunity for early prevention of chronic diseases including obesity.

Over recent decades, the dominance of ω-6 fatty acids in diets, particularly in industrialised countries, has led to suggestions that there maybe an association between the ratio of ω-6/ω-3 long chain polyunsaturated fatty acid (LCPUFA) and adipose tissue development during the critical early phases of life [11–13]. While ω-6 LCPUFA may have a stimulatory effect on fat cell development, there is evidence to suggest that ω-3 LCPUFA (DHA and EPA) have anti-obesity effects by decreasing lipid synthesis in cells [14–16]. Human adipose tissue starts to develop in the second trimester of pregnancy [17]. Fat cells acquired at an early stage in life may determine the level of fully differentiated adipocytes later in life, with only approximately 10% of fat cells being replaced across all ages and at all levels of BMI [18]. Collectively this evidence suggests that maternal intake of ω-3 fatty acids during pregnancy might help to reduce susceptibility to obesity later in life.

The effect of exposure to an increased supply of ω-3 LCPUFA in utero on body composition in the child has been examined in a number of RCTs. However, these have produced conflicting results. This systematic review and meta-analysis aimed to summarise the existing evidence from these RCTs, providing an update to the earlier systematic reviews on this topic [19–24] to include recently published trials.

## Methods

### Criteria for considering studies for this review

#### Types of studies

Only randomised controlled trials (RCT, including cluster randomised controlled trials and quasi-randomised controlled trials) with a minimum follow-up of 1 month postnatally were included. The review considered studies which reported body composition data and used maternal ω-3 LCPUFA supplementation. No language or country restrictions were applied.

#### Types of participants

Pregnant women and their offspring were considered as the target group for this systematic review. High-risk populations were not excluded.

#### Types of interventions

Trials were included that used ω-3 LCPUFA supplementation during pregnancy, irrespective of dose, formulation or mode of delivery and composition, e.g. oil, tablet. Trials were also included if the intervention(s) had been extended after pregnancy either during breast-feeding or directly to the infants or both.

#### Types of outcomes measures

Trials were included if they had reported measures of obesity or growth in the offspring, either as a primary or secondary endpoint. Obesity measures were defined as: body mass index (BMI), skin-fold thickness (SFT), obesity, overweight and fat mass. Growth measures (weight, height/length) were considered as secondary outcomes in the review. For trials in which obesity measures were assessed at more than one time point, only the results of the latest follow-up time point was included in the meta-analyses.

### Search strategy for identification of studies

A comprehensive search strategy, including all relevant synonyms for the main concepts, was developed covering the main bibliographic databases. Trials were identified through systematic searches within three main electronic databases, as advised by the Cochrane collaboration [25]:

- Cochrane Library (current issue) including:
  Cochrane Database of Systematic Reviews (CDSR)
  CENTRAL (trials)
  DARE
- MEDLINE (EBSCOhost)
- SCOPUS

When searching MEDLINE, the subject-specific terms were combined with the Cochrane Highly Sensitive Search Strategy for identifying randomised trials in MEDLINE: sensitivity-maximising version [25]. We adapted the preliminary search strategy for MEDLINE (EBSCOhost) for use in the other databases when relevant. The last search for literature was conducted in January 2018.

The clinical trials registry and WHO platform were searched for ongoing and recently completed trials. Conference proceedings were identified through the ISI Web of Science and, for retrieving theses the British Library E-Theses Online Service was searched. No language or publication status restrictions were imposed. References of included studies were crosschecked for additional studies.

### Data collection and analysis

#### Selection of studies

The main reviewer (MV) along with the second reviewer (HM) screened all the search results against the eligibility criteria and all those which were clearly irrelevant were excluded from further consideration. Thereafter, a tailored eligibility form was used by MV to appraise the retrieved studies, abstract and full text for relevance against the full inclusion criteria. Where there was uncertainty about inclusion of a particular study, this was discussed within the review team (MV, HM, TD) and a consensus was reached about the study eligibility. All the included studies were discussed and approved by the review team.

### Data extraction and management

MV extracted the data using a tailored data extraction form in EPPI Reviewer. Detailed information on study characteristics were recorded. Throughout the data extraction process, any disagreements about the interventions and outcomes were discussed and resolved within the review team. There was no blinding of the authors’ name, institutions, journals or the outcomes of the trials during the process. The extracted data were double checked by a second reviewer (HM) for accuracy against the trial reports.

### Assessment of risk of bias in included studies

The risk of bias tool described in the Cochrane Handbook for Systematic Reviews for Interventions was used to appraise the studies [26].

### Measurement of treatment effect

Dichotomous data were analysed as risk ratios or relative risk (RR) with 95% CI and continuous data as mean difference or standardised mean difference, with 95% CI.

### Unit of analysis issues

In trials with more than one intervention arm, multiple pairwise comparisons of intervention groups vs. comparator were avoided. Therefore, data from different intervention arms were pooled for an overall comparison with the control or placebo arm. The weight assigned to the control group was considered as the total number of participants in the comparator group vs. the total number of participants in the combined intervention arms [27].

### Dealing with missing data

All the relevant reported information for the number of missing participants was extracted and if undocumented, this was incorporated into the assessment of risk of bias. No imputed techniques were used for retrieving missing data.

### Assessment of heterogeneity

We used visual inspection of forest plots and the *χ*^2^ test to measure statistical heterogeneity between effect sizes of included studies. *I*^2^ statistics were used to quantify the amount of possible variability in effect estimates that is due to heterogeneity rather than chance (*I*^2^ < 25% low heterogeneity, ≥ 26% *I*^2^ < 74% moderate heterogeneity, *I*^2^ ≥ 75% high heterogeneity). Where there were heterogeneities between trials, a random effect model was used and meta-analysis reported if a moderate heterogeneity was found [25].

### Assessment of reporting biases

Every effort was made to identify unpublished studies through searching abstracts and ongoing trials databases. Publication bias was not assessed due to the small number of included studies [28].

### Data synthesis

We used EPPI Reviewer version 4.4.3.0. for conducting meta-analyses. Dichotomous data (events) and the number of participants were entered. We reported relative risk to describe the study effect [29].

### Subgroup analysis and investigation of heterogeneity

Where possible, sub-group analyses were performed for the control group and duration of follow-up.

### Sensitivity analysis

We did not conduct any sensitivity analysis because of the small number of studies that contributed to meta-analyses.

## Results

**Fig. 1:**
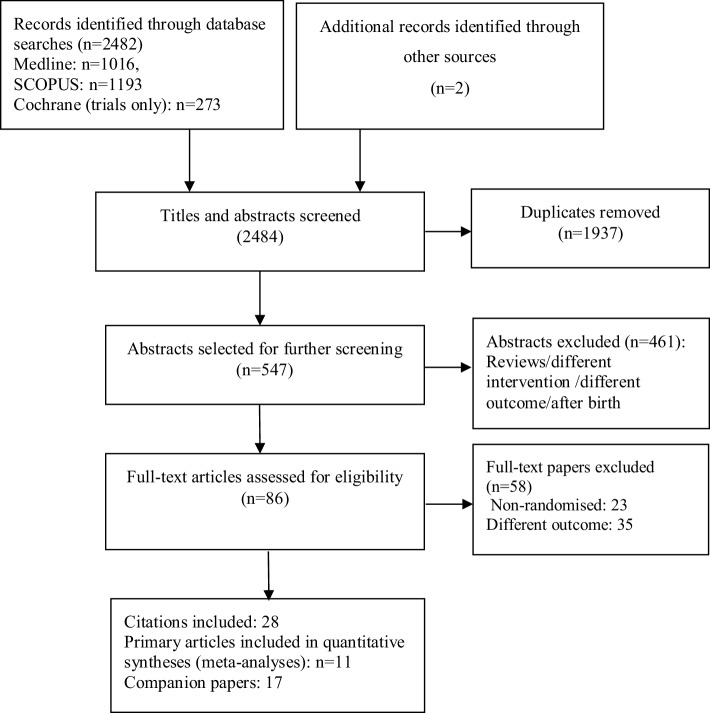
Study flow diagram, following preferred reporting items for systematic reviews and meta-analyses (PRISMA) criteria

Electronic searches yielded a total of 2484 results (Fig. 1). After removal of duplicates and non-relevant studies, the remaining 86 full text papers were assessed against the eligibility criteria for this systematic review. Eleven publications from ten unique RCTs were included in the final analyses, including a total of 3644 children. In the case of one trial [53], both the reports of an earlier [52] as well as latest follow-up data [54] were included. This was because the earlier report [52] included 1531 of children from the recruited sample in the original trial [53] whereas the later follow-up at 7 years [54] only included a sub-sample (a small percentage of pregnant women that were initially recruited at randomisation and assessed body composition outcomes in children using BOD POD and Bioelectrical Impedance Spectroscopy (BIS) methods). The characteristics of the included trials, their companion papers and study population are presented in Table 1. One trial was multi-centre (Germany, Hungry and Spain), four studies were conducted in Australia and Germany (two each) and the rest in Norway, Denmark, Mexico, the United States and Iran.

**Table 1:** Characteristics of included trials and study population for fatty acids and prevention of obesity in offspring

Primary article	Companion articles^a^	Country, enrolment period	Participants receiving intervention	Age at last F-U	Sample: high or low risk	Intake of Int. from/until	Duration of int. (months)	No. at randomisation^b^	No. at last F-U	Fatty acid product	Placebo	Daily dosage	Outcomes reported
Helland (2008) [31]	Helland et al. (2001) [30]	Norway, 1994–1996	Prenatally and postnatally-in mothers	7 years	Healthy women	18 weeks to 3 months postnatal	8–8.5	Pregnancy: 590 Birth: 341	143: 82 vs. 61	Cod liver oil (1183 mg/10 mL of DHA, 803 mg/mL of EPA)	Corn oil contained 4747 mg/10 mL of LA and 92 mg/10 mL of ALA	10 mL/day	BMI (kg/m^2^) Weight (kg) Height (cm)
Dunstan (2008) [34]	Dunstan et al. (2003, 2004) [32, 33]	Australia, 2000–2001	Prenatally	2.5 years	Women with allergic disease	20 weeks to birth	4.5–5	98 mothers	72: 33 vs. 39	Fish oil: 1.1 g EPA and 2.2 g DHA/day	Olive oil containing 2.7 g n-9 oleic acid	Four 1 g/day	Weight Height
Campoy (2011) [35]	Decsi (2005), Krauss-Etschmann et al. (2007), Escolano-margarit (2011) [36, 38, 37]	Germany, Spain, Hungary, 2001–2003	Prenatally and postnatally-in infants	6.5 years	Healthy women	22 weeks to 6 months postnatal	10–10.5	311 mothers	154: 37 vs. 37 vs. 35 vs. 45	Modified fish oil (500 mg DHA + 150 mg EPA) and Vit + Min	Fish oil + 5-MTHF^c^ OR 5-MTHF OR Placebo (nature not identified)	15 g one daily dose	BMI (kg/m^2^)
Rytter (2011) [39]	Olsen et al. (1992) [40]	Denmark, 1989–1990	Prenatally only	19 years	Non complicated pregnancies + no allergy to fish	30 weeks to delivery	2–2.5	533 mothers	243: 108 vs. 63 vs. 72	Fish oil (32% EPA, 23% DHA, together with 2 mg tocopherol/mL added to prevent auto oxidation of EPA and DHA)	Olive oil (72% oleic acid and 12% linoleic acid)	Four 1 g/day	BMI (kg/m^2^) Waist (cm) Insulin (pmol/L) Blood glucose (mmol/L) Hb A1c fraction (%) HOMA-IR Leptin (lg/L) Adiponectin (µg/L) IGF-I (µg/L) hs-CRP (mg/L)
Bergmann (2012) [41]	Bergmann et al. (2007, 2008) [42, 43]	Germany, Berlin, 2000–2002	Prenatally and postnatally-in mothers	6 years	Healthy pregnant Caucasian women	21–37 weeks and 3 months postnatal (optional)	4–7 (some women continued participation after delivery)	144 mothers	115: 41 vs. 74 (DHA vs. pooled control groups)	0.6 fish oil (200 mg DHA + 60 mg EPA)	(1) Basic Vit/Min (2) Prebiotic FOS^d^ (to promote a favorable gut flora)	0.6 g fish oil (200 mg DHA) + 4.5 g FOS + Vit/Min	Weight (kg) Height (cm) BMI (kg/m^2^) BMI *Z* score Sum of 4 SFT Head circumference
Gonzalez-Casanova (2015) [44]	Ramakrishnan et al. (2010), Stein et al. (2011) [45, 46]	Mexico, 2005–2007	Prenatally only	5 years	Non complicated pregnancies	From 18–20 weeks to delivery	5–5.5	1094 mothers	802: 403 vs. 399	Algal DHA	Corn-soy oil blend, not containing any DHA or other (n-3) fatty acids	400 mg (2 capsules taken at the same time)	BMI *Z* score Weight (kg) Height (cm) Height for age *Z* score Weight for age *Z* score
Brei (2016) [47]	Hauner et al. (2012), Brunner et al. (2013, 2015) and Much et al. (2013) [48, 49, 51, 50]	Germany, Munich, 2006–2009	Prenatally and postnatally-in mothers	5 years	Healthy women	15 weeks to 4 months postnatal	9.5–10 + 1 week	208 mothers	118: 61 vs. 57	N-3 LCPUFA [1020 mg (DHA) and 180 mg (EPA)]	Standard diet	1200 mg/day	Weight (kg) Head circumference Height (cm) BMI percentile Sum of 4 SFT Arm circumference Waist (cm) Ponderal index Biceps [mm] Triceps [mm] Subscapular Suprailiacal Body fat [%] Fat mass [g] Lean body mass [g] Subscapular/triceps-ratio Trunk-to-total SFT [%]
Muhlhausler (2016) [52]	Makrides (2010) [53]	Australia, Oct. 2005 to Jan. 2008	Prenatally only	5 years	Singleton pregnancies	< 21 weeks to delivery	15–19 weeks	1660 (enrolled at Adelaide centres)	1531: 770 vs. 761	DHA rich fish oil	Vegetable oil capsules without DHA	500 mg 3 capsule/day	Weight (kg) Head circumference Height (cm) Waist BMI BMI (*Z* score) Body fat (%) Total fat (kg)
Wood (2017) [54]	Makrides (2010) and Muhlhausler (2016) [53, 52]	Australia, Oct. 2005 to Jan. 2008	Prenatally only	7 years	Singleton pregnancies	< 21 weeks to delivery	15–19 weeks	504 from Adelaide centres that were not participating in the neurodevelopmental study	252: 132 vs. 118	DHA rich fish oil	Vegetable oil capsules without DHA	500 mg 3 capsule/day	Weight (kg) Height (cm) Waist BMI BMI (*Z* score) Body fat (%) Fat mass (kg) Hip
Foster (2017) [55]	–	USA	Prenatally only	4 years	Obese or with gestational diabetes	25–29 weeks to delivery	13–17 weeks	72	69: 34 vs. 29	DHA	Corn/soy oil	800 mg/day	Weigh *Z* score Height *Z* score BMI *Z* score Arm skinfold Arm circumference
Ostadrahimi (2017) [56]	Ostadrahimi (2017 [57]	Iran	Prenatally only	6 months	Singleton low-risk pregnancies	16–20 weeks to 1 month after birth	26–30 weeks	150	145: 75 vs. 71	Fish oil (120 mg DHA, 180 mg EPA)	Liquid paraffin	1000 mg	Weight (kg) Length (height) (cm) Head circumference

a Published data and conference presentations, no unique data were extracted from conference abstracts

b Indicates the number at randomisation, where recruitment has occurred prenatally

c MethylTetraHydroFolic

d Fructo-oligosaccharide

### Study design

All the included trials were parallel RCTs and most trials had two parallel groups while two had three parallel groups, either as intervention or control. The study by Campoy et al. [35] defined interventions as modified fish oil plus vitamin and mineral, fish oil plus 5-methyltetrahydrofolic (MTHF) and 5-MTHF only. In this systematic review, we have only considered modified fish oil component plus vitamin and mineral as the intervention arm compared to placebo. Rytter and colleagues [39] also had two control groups, ‘olive oil’ or ‘no oil’. Olive oil was used as the comparator for meta-analyses in this systematic review. In addition, the nature of the control group (standard diet) in the study by Brei et al. [47] was different from other trials and, therefore, this study was included in the sub-group analyses.

It is important to note that most of the trials were primarily designed to investigate other outcomes in children such as neurological development, maternal insulin sensitivity; and growth measures in the offspring are also reported as secondary end-points [31, 34, 35, 39, 41, 44, 52, 54, 55]. Only two trials [47, 56] were originally designed to evaluate the effectiveness of prenatal n-3 LCPUFA supplementation on infant’s body composition. The study by Brei et al. [47] was an open label trial and applied a combined intervention approach of fish oil capsules and arachidonic acid-balanced diet (ratio of ω-6/ω-3) during pregnancy. This study also had a high non-participation rate at the 5 years follow-up. In the study by Ostadrahimi et al. [56], the primary outcome was defined as infants’ neurodevelopment status and anthropometric measures were reported as secondary end-points.

### Participants and sample sizes

One trial was conducted in atopic women [34] and another in women who were obese or had a history of gestational diabetes [55]. The remainder of the trials involved healthy pregnant women with non-complicated pregnancies. Studies varied in their sample size at randomisation from 72 [55] to 1660 [52] pregnant women.

### Intervention

With the exception of one study that used algal DHA [44], all other studies used either DHA alone or a combination of EPA and DHA together. In the study by Campoy et al. [35] vitamin and mineral supplements were also used along with the fatty acid component. Furthermore, Brei et al. [47] provided women with ω-3 LCPUFA intervention as well as nutritional counseling, focused on reducing the consumption of n-6 fatty acid (AA) to a moderate level of intake (90 mg AA per day). Six trials supplemented women during pregnancy only and in the case of four trials [32, 35, 47, 56] the intervention was continued postnatally with various durations.

Compliance with the intervention was assessed by a variety of methods, including the number of fatty acids capsules ingested, divided by the number the participant should have ingested multiplied by 100; standardised questionnaires at gestation; the percentage of the total number of capsules expected to be consumed; and measuring fatty acids levels in erythrocytes at 30 and 37 gestational week, and 6 weeks postnatally. Four studies did not report the method of measuring adherence to the intervention [32, 41, 47, 56].

### Reported obesity outcomes and follow-up duration

Anthropometric measurements were conducted using standardised references, e.g. WHO reference ranges (including BMI-Z) and by means of standard tools. The definition and diagnosis method for each outcome included in meta-analyses is presented in Table 2. The duration of follow-up in trials ranged from 6 months [56] to 19 years [39]. The most frequently reported obesity measures were BMI and the most reported growth measures were weight and height.

**Table 2:** Definition of obesity measures included in the meta-analyses and their measurement in individual studies

Studies	Measurement
Helland (2008) [31], 7 years follow-up	
BMI	Not defined
Weight	Not defined
Height	Not defined
Dunstan (2008) [34], 2.5 years follow-up	
Weight	Not defined
Height	Not defined
Campoy (2011) [35], 6.5 years follow-up	
BMI	Not defined
Rytter (2011) [39], 19 years follow-up	
BMI	Not defined
Bergmann (2012) [41], 6 years follow-up	
BMI	Length (height) and weight were measured using Harpenden measuring boards, small measuring tapes and calibrated Seca balances and BMI was calculated accordingly
BMI-Z	BMIs of children from birth to 6 years were standardised with age-specific means of the World Health Organisation (WHO) multi-centre growth reference study
Weight	Using calibrated Seca balances
Height	Harpenden measuring boards and small measuring tapes
Sum of SFT	Using a Holtain caliper at the midtricipital, the subscapular and the suprailiac measuring point
Gonzalez-Casanova (2015) [44], 5 years follow-up	
BMI-Z	BMI was computed by calculating age at measurement from the date of birth and then converted to age-specific *Z* scores using the 2006 WHO reference standards
Weight	Using a Tanita scale to the nearest 10 g
Height	Using a Seca stadiometer to the nearest 1 mm
Brei (2016) [47], 5 years follow-up	
Weight	Using a standard flat scale (Seca Clara), to the nearest 100 g with the child in a standing position
Height	Using a stadiometer, to the nearest 0.5 cm with the child in a standing position
Sum of SFT	SFTs were measured in triplicate with the use of a Holtain caliper (Holtain Ltd.) at 4 different body sites on the left body axis (triceps, biceps, sub- scapular, and suprailiac), at the study center or at the family’s home. The mean of the 3 measurements was used for the SFT value, and the sum of the 4 SFTs was calculated
Body fat (%)	Using predictive skinfold regression equations according to Weststrate and Deurenberg
Fat mass (kg)	Using predictive skinfold regression equations according to Weststrate and Deurenberg
Muhlhausler (2016) [52], 5 years follow-up	
BMI	Weight using electronic scales (without shoes and in underwear to the nearest 100 g); height using a stadiometer without shoes and BMI was calculated accordingly
BMI-Zs	The BMI measures for each child were compared with standardized reference charts for the child’s age and sex to calculate their *Z* scores
Weight	As above
Height	As above
Body fat (%)	Using the equation as: [fat-free mass − body weight|body weight] × 100
Fat mass (kg)	Using bioelectrical impedance spectroscopy
Foster (2017) [55], 4 years follow-up	
BMI-Z	Each child was weighed on a digital scale with no shoes and measured to the nearest 0.1 kg with the procedure repeated. The two weight measurements were required to be within 0.2 kg for accuracy and precision. Standing height was measured in cm on a stadiometer with a fixed vertical bar and an adjustable headpiece with shoes removed. Height measurement was recorded to the nearest 0.1 cm, and then the process was repeated. The two height measurements were required to be within 0.2 cm for accuracy and precision. *Z* scores were calculated using the Centers for Disease Control and Prevention (CDC) reference data
Ostadrahimi (2017) [57], 6 months follow-up	
Weight	Using a lever scale with a precision of 0.1 kg (Seca, Germany)
Height	Using a stadiometer table accurate to 0.1 cm in the supine position without shoes and hats

Also, while the trial by Wood [54] reported longer-term follow-up at 7 years from the original study [53], data from the earlier follow-up at 5 years were included in meta-analyses. This was because only 30.3% of the original sample recruited in the Adelaide centres (504 of 1660 pregnant women that were not participating in the neurodevelopmental study) were eligible to participate in the body composition follow-up study at 7 years [54]. Of the 504 women invited only 252 [50%] consented to participate and therefore, the sample may not be representative of the original recruited women. The results from this study [49] are described narratively.

### Quality of RCTs

**Fig. 2:**
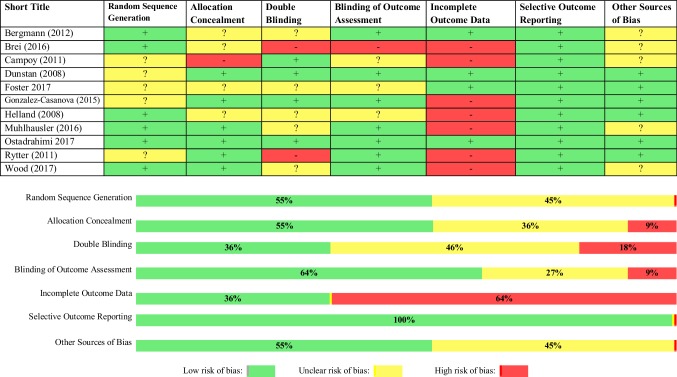
Risk of bias assessment figure in the included trials

The methodological quality of the included trials varied as shown in Fig. 2. Six trials had a low risk of bias in both their random sequence generation and allocation concealment (55%) in each domain. Blinding of both participants/staff and outcome assessment were deemed as low risk of bias in four and seven trials, respectively. Over half of the trials were rated as high risk of bias for the completeness of data (64%) because of high attrition bias. All trials were deemed to have a low risk of bias for the reported outcomes and five trials were rated as unclear for other potential sources of bias.

### Pooled effect of interventions

The definition of the outcomes in each study, as included in meta-analyses, and their diagnosis method are presented in Table 2.

### BMI as outcome measure

**Fig. 3:**
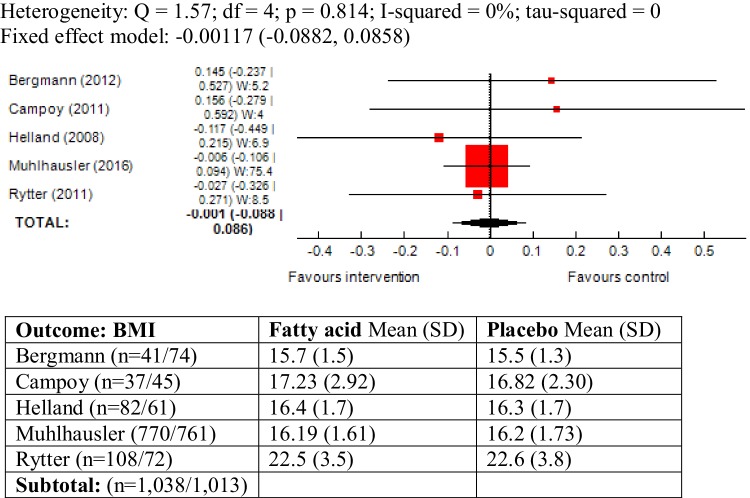
Forest plot for prenatal intake of fatty acid vs. placebo for BMI

Five trials reported BMI in children and statistically, the studies were largely homogenous (*χ*^2^ = 1.57, *P* = 0.81, *I*^2^ = 0%). The pooled results did not show an association between maternal ω-3 LCPUFA intervention during pregnancy and BMI in the offspring (mean difference (SMD) − 0.001, 95% CI − 0.08, 0.08; 2051 children) (Fig. 3).

A sub-group analysis was also conducted including only the studies with comparable duration of follow-up [32, 35, 41]. The results did not yield any significant change (SMD 0.001, 95% CI − 0.089, 0.92; 1871 children) (Forest plot not shown).

### BMI-Z as outcome measure

**Fig. 4:**
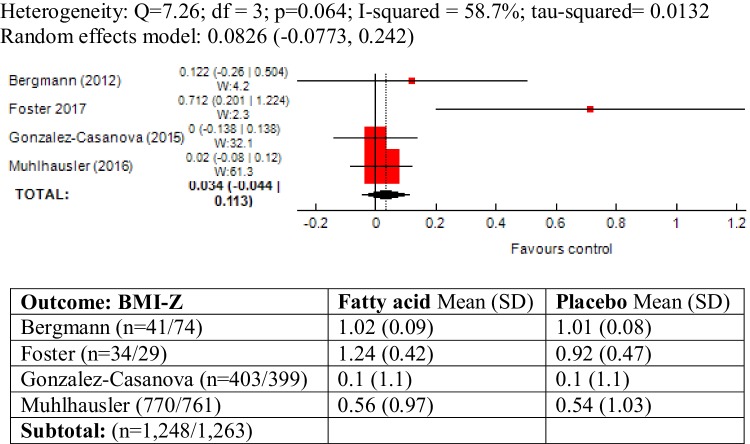
Forest plot for prenatal intake of fatty acid vs. placebo for BMI-Z

The outcome was reported in four trials and moderate heterogeneity was observed between the trials (*χ*^2^ = 7.26; *P* = 0.06; *I*^2^ = 58.7%). Meta-analysis of these trials did not show an association between maternal ω-3 LCPUFA intervention during pregnancy and BMI-Z in offspring (SMD 0.082, 95% CI − 0.077, 0.24; 2511 children) (Fig. 4).

The study by Foster [55] recruited a high-risk sample of pregnant women, with obesity or a history of gestational diabetes and excluding this study from meta-analysis there was no heterogeneity between trials (*I*^2^ = 0%). However the pooled results did not significantly change (SMD 0.0178, 95% CI − 0.0616, 0.0972) (Forest plot not shown).

### Sum of SFT as outcome measure

**Fig. 5:**
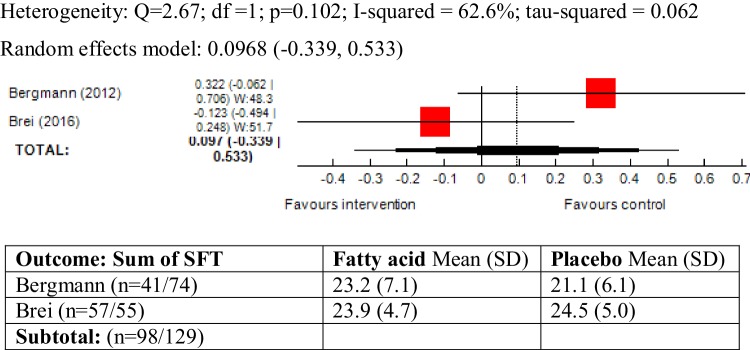
Forest plot for prenatal intake of fatty acid vs. placebo for sum of SFT

Skinfold thickness was reported in three trials and the pooled results showed a high level of heterogeneity between trials (*χ*^2^ = 38.6, *P* = 4.06, *I*^2^ = 94.8%). The study by Foster [55] only reported a single measure of arm SFT, as opposed to the sum of SFT for 3–4 body points reported in other trials, and given that the study was also conducted in high-risk pregnant women (obese/history of gestational diabetes), we pooled the results excluding this study from the meta-analysis. A moderate level of heterogeneity was found between the two trials (*χ*^2^ = 2.67; *P* = 0.10, *I*^2^ = 62.6%) and the meta-analysis did not show an association between maternal ω-3 LCPUFA intervention during pregnancy and sum of SFT in the offspring (SMD 0.09, 95% CI − 0.33, 0.53, 227 children) (Fig. 5).

### Body fat (%) and fat mass (kg) as outcome measure

**Fig. 6:**
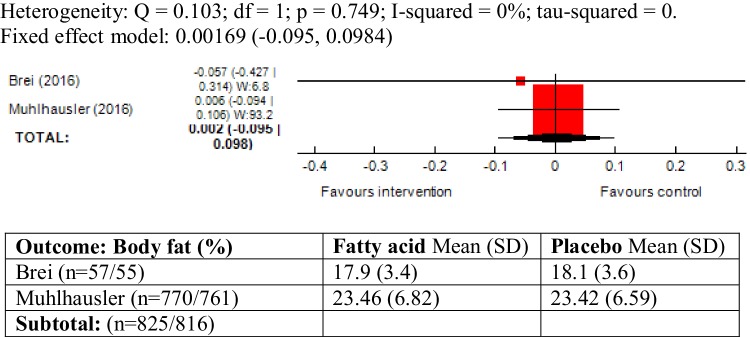
Forest plot for prenatal intake of fatty acid vs. placebo for body fat (%)

**Fig. 7:**
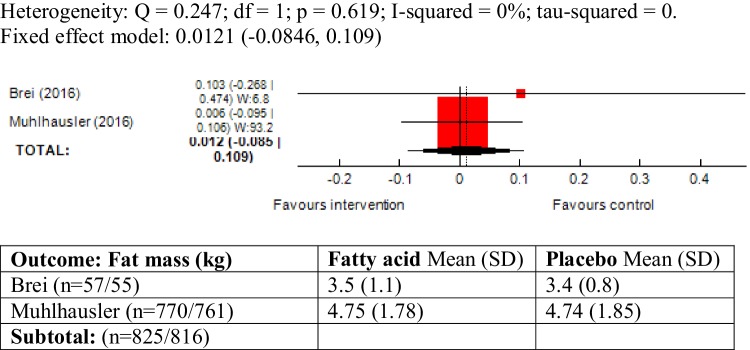
Forest plot for prenatal intake of fatty acid vs. placebo for fat mass (kg)

There was no heterogeneity between the two trials reporting these outcomes (body fat (%): *χ*^2^ = 0.10; *P* = 0.74, *I*^2^ = 0% and fat mass: *χ*^2^ = 0.24; *P* = 0.61, *I*^2^ = 0%). The meta-analyses did not show an association between maternal ω-3 LCPUFA intervention during pregnancy and body fat (%) (SMD 0.00, 95% CI − 0.09, 0.09; 1641 children) and fat mass (kg) (SMD 0.01, 95% CI − 0.08, 0.10; 1641 children) in the offspring (Figs. 6, 7).

### Weight as outcome measure

**Fig. 8:**
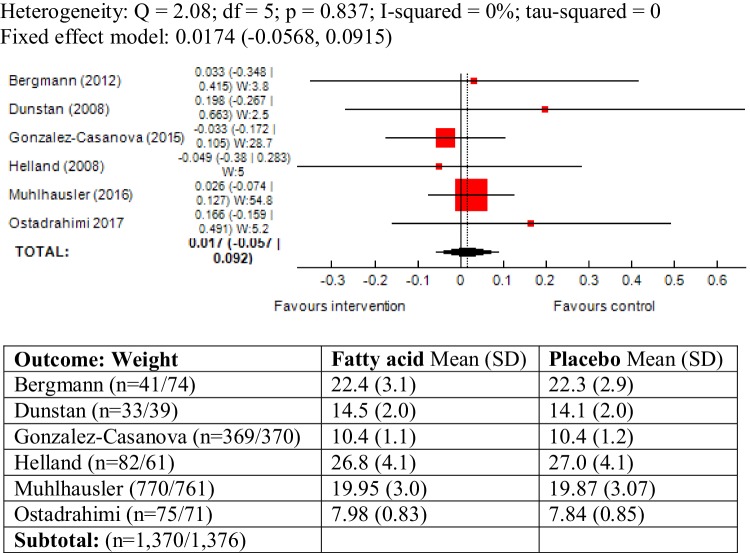
Forest plot for prenatal intake of fatty acid vs. placebo for weight

The outcome was reported in six trials and there was no heterogeneity between studies (*χ*^2^ = 2.08; *P* = 0.83, *I*^2^ = 0%). The results of meta-analysis did not show an association between maternal ω-3 LCPUFA intervention during pregnancy and weight in offspring (SMD 1.01, 95% CI − 0.056, 0.091; 2746) (Fig. 8).

A sub-group analysis was also conducted including the study conducted by Brei et al. [47], which defined “standard diet” as its comparator. The studies were largely homogenous (*I*^2^ = 0%) and the meta-analysis did not alter the results greatly (SMD 0.026, 95% CI − 0.046, 0.099) (Forest plot not shown).

In the study by Ostadrahimi children were followed-up by 6 months of age [56] and thus, weight was reported as a growth measure. Since the study had the shortest follow-up duration compared to the other included trials, a meta-analysis was conducted excluding this study and did not alter the results (SMD 0.009, 95% CI − 0.0669, 0.0854).

### Height as outcome measure

**Fig. 9:**
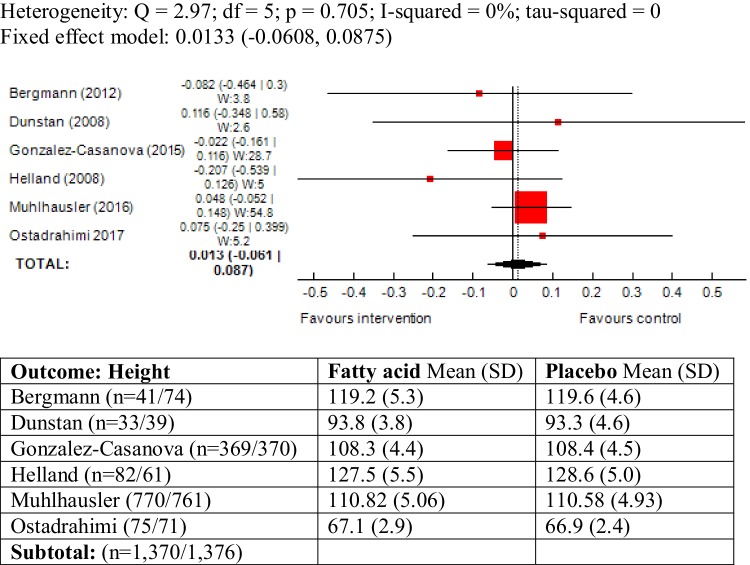
Forest plot for prenatal intake of fatty acid vs. placebo for height

The outcome was reported in six trials and statistically, there was no heterogeneity between studies (*χ*^2^ = 2.97; *P* = 0.70, *I*^2^ = 0%). The results of meta-analysis did not show an association between maternal ω-3 LCPUFA intervention during pregnancy and height in offspring (SMD 0.01, 95% CI − 0.06, 0.08; 2746) (Fig. 9).

A sub-group analysis was also conducted including the study conducted by Brei et al. [47], which defined “standard diet” as its comparator. The studies were largely homogenous (*I*^2^ = 0%) and the meta-analysis did not alter the results greatly (SMD 0.025, 95% CI − 0.046, 0.098) (Forest plot not shown).

In the study by Ostadrahimi children were followed-up by 6 months of age [56] and thus, height was reported as a growth measure. Since the study had the shortest follow-up duration compared to the other included trials, a meta-analysis was conducted excluding this study and did not alter the results (SMD 0.009, 95% CI − 0.066, 0.086).

### Weight *Z* score and height *Z* score as outcome measures

Two trials by Gonzalez-Casanova et al. [44] and Foster et al. [55] reported these outcomes and pooling the results, the trials were largely heterogeneous (weight *Z* score: *χ*^2^ = 11; *P* = 0.00, *I*^2^ = 90.9% and height *Z* score: *χ*^2^ = 8.61; *P* = 0.003, *I*^2^ = 88.4%). There were no associations between maternal ω-3 LCPUFA intervention during pregnancy and weight *Z* score and height *Z* score (Forest plots not shown).

### Description of the outcomes reported in Wood et al. [54] study

This study reported body composition results at 7 years follow-up in a sub-sample of total recruited pregnant women in Adelaide centres from the original study [53]. Body composition measurements were assessed using two methods of Air Displacement Plethysmography (BOD POD system) and BIS (Table 3). The results suggest that the maternal ω-3 LCPUFA intervention during pregnancy did not have an influence on childhood anthropometric and body composition measures at 7 years follow-up.

**Table 3:** List of the reported clinical outcomes in Wood 2017 study [54]

	DHA (*n* = 132) Mean (SD)	Control (*n* = 118) Mean (SD)	*P* value
BMI	16.48 (2.3)	16.25 (2.56)	0.46
BMI-Z	0.43 (1.07)	0.25 (1.19)	0.22
Weight (kg)	25.18 (4.52)	25.36 (5.31)	0.77
Height (cm)	123.3 (5.11)	124.6 (5.71)	0.07
Body fat (%), BOD POD	19.19 (6.89)	19.61 (7.63)	0.58
Body fat (%), BIS	24.22 (7.42)	22.78 (6.68)	0.11
Fat mass (kg), BOD POD	5.2 (3.0)	5.12 (3.33)	0.86
Fat mass (kg), BIS	6.28 (2.9)	5.99 (3.18)	0.46

## Discussion

Eleven RCTs were identified, with a total number of 3644 children whose mothers started supplementation with ω-3 LCPUFA during pregnancy and for whom obesity and growth measures were followed-up during childhood. Trials were heterogeneous for their sample, reported outcomes and duration of follow-up. Random sequence generation was deemed to be adequate in six of the included trials. Two and five trials were also judged to have a high and unclear risk of bias respectively for their performance bias and additionally, seven trials had a high attrition bias. The findings from this systematic review and meta-analyses did not provide evidence that intake of ω-3 LCPUFA during pregnancy could protect against subsequent development of a number of childhood obesity outcomes i.e. BMI, BMI-Z, weight, height, sum of SFT, body fat (%) and fat mass (kg). Overall, the results from this review need to be considered with caution because of the large heterogeneities observed between studies and also in light of the assessment for risk of bias. It is also worth mentioning that only two trials [47, 56] were initially designed to assess growth and obesity measures in children following maternal ω-3 LCPUFA supplementation during pregnancy. The results are consistent with those of the meta-analyses; neither found a significant difference between the study arms for growth measures at 6 months [56] and obesity measures at 5 years [47] follow-ups.

### Overall completeness and applicability of evidence

There was no evidence that ω-3 LCPUFA supplementation during pregnancy is effective for prevention of obesity in the offspring. The heterogeneity between the trials limited the findings and where statistical heterogeneities exist, random effect models were used to pool the results. Heterogeneity between studies originated from variations in: dosage and timing of intervention, comparators, methods for reporting the outcome measures across studies, locations/settings and the follow-up duration. One study [39] also used DHA, isolated from algae, which may act differently from fish oil [58]. It was not possible to conduct stratified analyses for the type, dosage and timing of intervention to explore the differences due to the relatively small number of trials in each group. In addition, although most studies recruited a relatively large sample, only a small number of participants agreed to participate or could be approached at follow-up assessments.

### Quality of evidence

Overall, the trials were at moderate to high risk of bias (Fig. 2). Randomisation and allocation concealment were deemed as unclear for 45% and 36% of included studies respectively. High loss to follow-up was also a major concern where 64% of studies showed high attrition which might have largely biased the effect of intervention within these trials. In addition, not many studies have reported these outcomes in the context of maternal ω-3 LCPUFA intervention during pregnancy. Also, measures of obesity and growth such as BMI and weight were the most reported outcomes in trials and the use of more precise methods for measurement of obesity in children such as MRI/ultrasound techniques and skinfold thickness, as sum or single measure, were reported in only a few trials [41, 47].

### Strengths and limitations

This systematic review only included trials in which maternal ω-3 LCPUFA supplementation was commenced during pregnancy, thus, crucially, enabling the effect of ω-3 LCPUFA intervention during pregnancy for prevention of childhood obesity to be isolated. Also following an a priori published protocol, a comprehensive search strategy allowed for a complete coverage of all the relevant literature through citation databases, trial registries and conference proceedings with no limitation by language or publication status. Moreover, a range of obesity and growth measures were the focus of the present review and the most up-to-date results from the trials, reported as the longest available follow-up data, are included in the meta-analyses. A limitation of the review is that sub-group analyses were not conducted as planned e.g. duration of intervention, dosage of ω-3 LCPUFA because of the limited number of studies that could contribute in the meta-analyses.

### Consistency with other reviews

Although similar systematic reviews have been conducted, they have either examined fatty acid supplementation during the prenatal period and also exclusively in breastfeeding [23, 24] or have only examined outcomes at birth [22]. Also, other reviews on this topic have narratively described the effectiveness of prenatal and postnatal supplementation with ω-3 LCPUFA on an infant’s body composition [19–21]. The review by Stratakis et al. [23] conducted meta-analyses only on BMI for different age groups, although the review by Li et al. [24] assessed a wide range of obesity measures in children e.g. BMI, weight, sum of SFT.

The main distinction of this systematic review is that it examined intake of ω-3 LCPUFA commenced during pregnancy and (where applicable) continued postnatally, examining a wide range of obesity and growth measures. The findings from this systematic review did not support the hypothesis that maternal ω-3 LCPUFA could protect against obesity-related measures in offspring. We have also reported methodological shortcomings including number of studies, small sample sizes and attrition bias.

## Authors’ conclusion

### Implications for practice

The results of the current systematic review do not provide an evidence for the prevention of obesity in the offspring by maternal ω-3 LCPUFA intervention during pregnancy when compared with placebo/no treatment. Due to the high heterogeneity between studies along with small sample size and large attrition at follow-ups, the effects of ω-3 LCPUFA supplementation during pregnancy for prevention of childhood adiposity in the long-term remains unclear.

### Implications for research

While the meta-analyses conducted as part of this systematic review did not support the effectiveness of ω-3 LCPUFA supplementation during pregnancy and lactation on childhood obesity, the heterogeneity in all aspects of the included trials makes it difficult to determine whether this is due to the lack of an effect, or differences in study design. Hence, the effect of maternal ω-3 LCPUFA intervention during pregnancy for prevention of childhood obesity need to be further investigated in large, high quality RCTs. Further research need also to determine specific sub-groups in the population that might get a greater benefit and whether different omega-3 LCPUFA i.e. EPA vs. DHA might potentially have different effects.

Given that only two included studies had been established to explicitly examine the effect of maternal ω-3 LCPUFA intervention during pregnancy on obesity in offspring, such RCTs need to be designed specifically to examine this question. Trials should also consider the effect of ω-6/ω-3 ratio in the dietary intervention and, rather than increasing ω-3 LC-PUFA in isolation, to determine the role of the balance of fatty acid intake in maternal diet. Using combined methods of anthropometric and SFT measurement as well as more precise measures such as MRI and ultrasound will also allow for more accurate estimation of adipose tissue deposition in children.

The optimal timing of ω-3 LCPUFA intervention in pregnancy is another key factor that needs to be further investigated. The first appearance of adipocytes in the human foetus occurs in second trimester of pregnancy, between 14 and 16 weeks of gestation [17]. Further research is required to determine the critical window for programming of offspring adipose tissue. Baseline level of DHA in pregnant women, type and optimal dose of ω-3 LCPUFA, as well as the choice of control regimens, are elements that need to be considered in further trials. More importantly, additional rigorous strategies are needed to minimise the low participation rate at follow-up assessments. Recruiting fewer participants or high attrition rates in interventions with ω-3 PUFAs could lead to significantly varied findings and thus bias in impact of fatty acids, as highlighted in a recent study [59]. Therefore, longitudinal studies with adequate sample size and repeated measurements are required to provide strong evidence with which to determine the effect of ω-3 LCPUFA intake during pregnancy on obesity in offspring. Furthermore, the majority of the studies were conducted in developed countries; it remains a priority to also understand the effectiveness of these interventions for childhood obesity among underreported populations.
